# Plasma miR-21, miR-155, miR-10b, and Let-7a as the potential biomarkers for the monitoring of breast cancer patients

**DOI:** 10.1038/s41598-018-36321-3

**Published:** 2018-12-19

**Authors:** Solmaz Khalighfard, Ali Mohammad Alizadeh, Shiva Irani, Ramesh Omranipour

**Affiliations:** 1 Department of Biology, Science and Research Branch, Islamic Azad University, Tehran, Iran; 2 Cancer Research Center, Tehran University of Medical Sciences, Tehran, Iran; 3 Breast Disease Research Center, Tehran University of Medical Sciences, Tehran, Iran

## Abstract

There is a pressing need for further studies to categorize and validate circulating microRNAs (miRs) in breast cancer patients that can be one of the novel strategies for cancer screening and monitoring. The present study is aimed to investigate the expression of the circulating candidate microRNAs after the operation, chemotherapy, and radiotherapy in the non-metastatic breast cancer patients. Tumor tissue and plasma samples were collected from the 30 patients with recently diagnosed Luminal A breast cancer. Control plasma samples were collected from the 10 healthy subjects. A panel of four miRs including miR-21, miR-55, miR-10b, and Let-7a were selected and their expression levels were measured before and after the operation, chemotherapy, and radiotherapy by using Real-Time PCR technique. The plasma expression of the miR-21, miR-155, and miR-10b was significantly increased and the Let-7a plasma expression decreased in the breast cancer patients compromised to the control ones. There was a similar expression pattern of the miRs between the tissue and plasma samples. The plasma levels of the miR-21, miR-155, and miR-10b were significantly down-regulated and the Let-7a plasma level was up-regulated after the operation, chemotherapy, and radiotherapy compromised to the pre-treatment. There was a significant difference in the miR-155 plasma level after the operation, chemotherapy, and radiotherapy compromised with each other. Moreover, there was no significant difference between the plasma levels of the miRs after the radiotherapy compromised to the control cases. The operation, chemotherapy, and radiotherapy led to a more reduction in the oncomiRs and an increase in the tumor suppressor-miRs. It seems that monitoring miRs during treatment might be considered as a respectable diagnostic tool for monitoring of breast cancer patients.

## Introduction

Breast cancer (BC) is the second leading cause of gynecological cancer deaths^1^. The diagnosis of BC in the early stages, as well as monitoring of the disease progression and response to treatment, could be made easy with the aims of the liquid biopsy in the neoadjuvant setting^2^. In this respect, existing diagnostic tools and biomarkers for BC have many inherent deficiencies^3^. A number of the circulating tumor markers including carcinoembryonic and carbohydrate antigens are widely used in BC monitoring, but the sensitivity of these markers is low^3^. Therefore, they cannot be used as reliable screening tools, although they have long been in clinical approaches. An ideal biomarker should be easily accessible such that it can be sampled noninvasively and be sensitive enough to detect the early presence of tumors^4^. This new approach has the potential to revolutionize clinical management including determining cancer classification, estimating prognosis, predicting therapeutic efficacy, maintaining surveillance following surgery as well as forecasting disease recrudescence^5^.

MicroRNAs (miRs) are the short single-stranded RNAs that have known as important regulators of the various cellular processes^6^. An estimated 30–60% of the genome is regulated by miR-mediated silencing^6^, though the aberrant expression of the miRs is associated with many diseases such as cancer. Early studies showed that some miRs can regulate cellular differentiation, proliferation and apoptosis processes that can be important in the cancer aggravation. A number of the differentially derived miRs from the tissues have reported and their expression profiles may be used as the potential biomarkers for the diagnosis, prognosis, and therapy. In addition, the discovery of the roles of the miRs in developing BC may provide new opportunities for the development of the novel strategies for diagnosing and treating this type of the malignancy.

To date, only a few studies have begun to profile the circulating miRs in blood^7^. The analysis of the circulating miRs is at an early stage of development, and there is a persuasive need for further studies to categorize and validate circulating miR biomarkers in BC. Circulating miRs have been found to be significantly elevated in the blood of the cancer patients compromised with the healthy controls^8^. Furthermore, the elimination of the primary tumor leads to the loss of raised circulating miRs; suggesting that many of the miRs are ‘tumor-derived’ and ‘cancer-specific’. The current belief is that these ‘tumor-derived’ circulating miRs are released from the primary tumor via the exosome vesicles and apoptotic bodies, though the discoveries of the exact underlying mechanisms are still developing^9^. Blenkiron *et al*.^10^ detected the expression levels of the different miRs between the basal and Luminal subtypes of the 309 miRs in 93 breast cancer patients^10^. Miska *et al*. (2015) showed that the different molecular subtypes of the BC including Luminal A, Luminal B, Basal-like, HER2, and Normal-like present the expression profiles of the different miRs^11^. Moreover, a set of miRs was able to classify Luminal A from Luminal B tumors with the high expression of the Let-7 family members with Luminal A tumor, ER-positive status, and low tumor grade (Table 1)^12^.

**Table 1:** Main oncogenic and tumor suppressive of miRNAs in Luminal A breast cancer.

MicroRNAs	Target genes
**Up-regulation**	
Mir-181	ATM
Mir-155	SOCS1
Mir-10b	HOXD10
Mir-373	CD44
Mir-520	CD44
Mir-103	DICER
Mir-107	DICER
Mir-21	MASPIN, TPM1, PDCD4
Mir-31	FZD3, ITGA5, RDX, RHOA
Mir-193b	UPA
Mir-221	P27
Mir-222	P27
Mir-125b	BAK1
**Down-regulation**	
MIR-30e	ITGB3, UBC9
Mir-200	HAS, TUP, CFA
Let -7	HAS, TUP, CFA, SSC, BTA, MMU
Mir-335	SOX4, TNC
MIR-126	SSC, BTA, MMU
Mir-206	TUP, CFA, SSC,
MIR-451	MDR1
Mir-345	MRP1
Mir-7	MRP1
Mir-125b	EPO, EPOR, ENPEP, CK2-α,CCNJ, MEGF9, ERBB2
Mir-205	HMGB3
Mir-17–92	Mekk2
Mir-146	NFkB, STAT3
Mir-31	RhoA, WAVE3, RhoA, WAVE3

In cancer, miRs can be classified as oncogenes (oncomiRs) or tumor-suppressor miRs^13^. Chromosomal regions of the encompassing oncogenic miRs may be amplified that result in the increased expression of the oncomiR such as miR-21, miR-155, and miR-10b and the decreased the tumor suppressors such as Let-7a^14^. The Let-7a displays a lower expression in cancer cells and can suppress oncogene expression, thereby, may control the cellular differentiation^15^. In this respect, the distinct biological properties of the miRs including the remarkable stability, accessibility for rapid and accurate quantification, and a direct link with disease states make them the ideally suitable to serve as the minimally invasive biomarkers to track the disease^16^. Based on these data, in the first section of the present study, the correlation of the expression level of several dis-regulated miRs was assayed between the tissue and plasma samples in the breast cancer patients. In the second section, we explored the influence of the tumor resection and chemo-radiation on the expression of the candidate miRs and presented discussions with an emphasis on their role in monitoring treatment responses.

## Results

### Pre-treatment analysis of the candidate miRs

**Figure 1:**
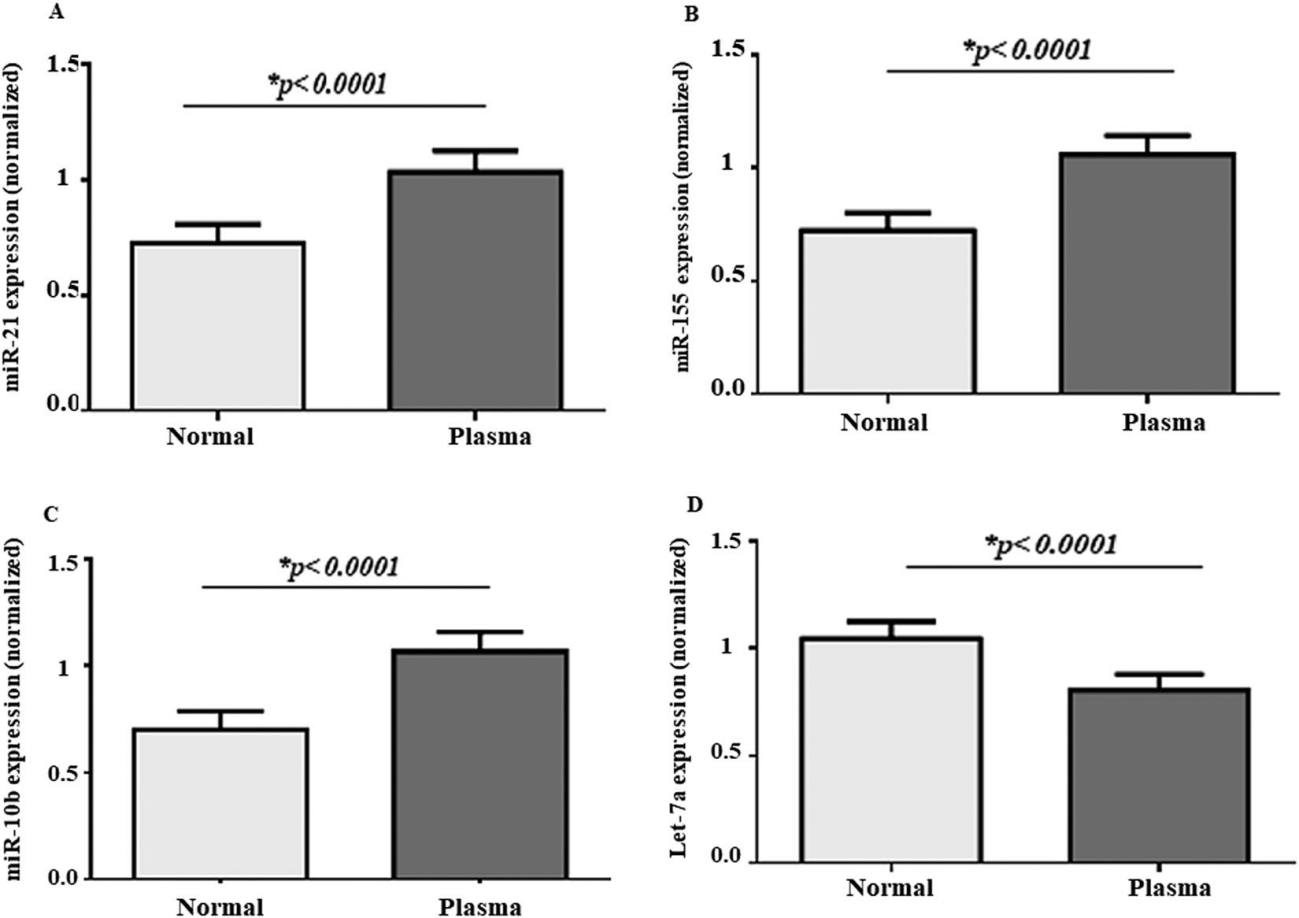
Comparison of the selected plasma miRs between the breast cancer patients and control. The relative expression level of the miR-21 (**A**), miR-155 (**B**), miR-10b (**C**), and Let-7a (**D**) were normalized using SNORD RNA as reference RNA.

We have determined the expression of miR-21, miR-10b, miR-155, and Let-7a in the plasma and the tissue of 40 samples (30 from patients with breast cancer and 10 from the controls). Except for three patients in the radiotherapy, all cases received cycles of the surgery, chemotherapy, and radiotherapy during the course of the study. Consequently, the relative abundance of the miR-21, miR-10b, and miR-155 was significantly up-regulated, and the Let-7a expression was significantly down-regulated in the plasma of the cases compromised with the control (Fig. 1).

**Figure 2:**
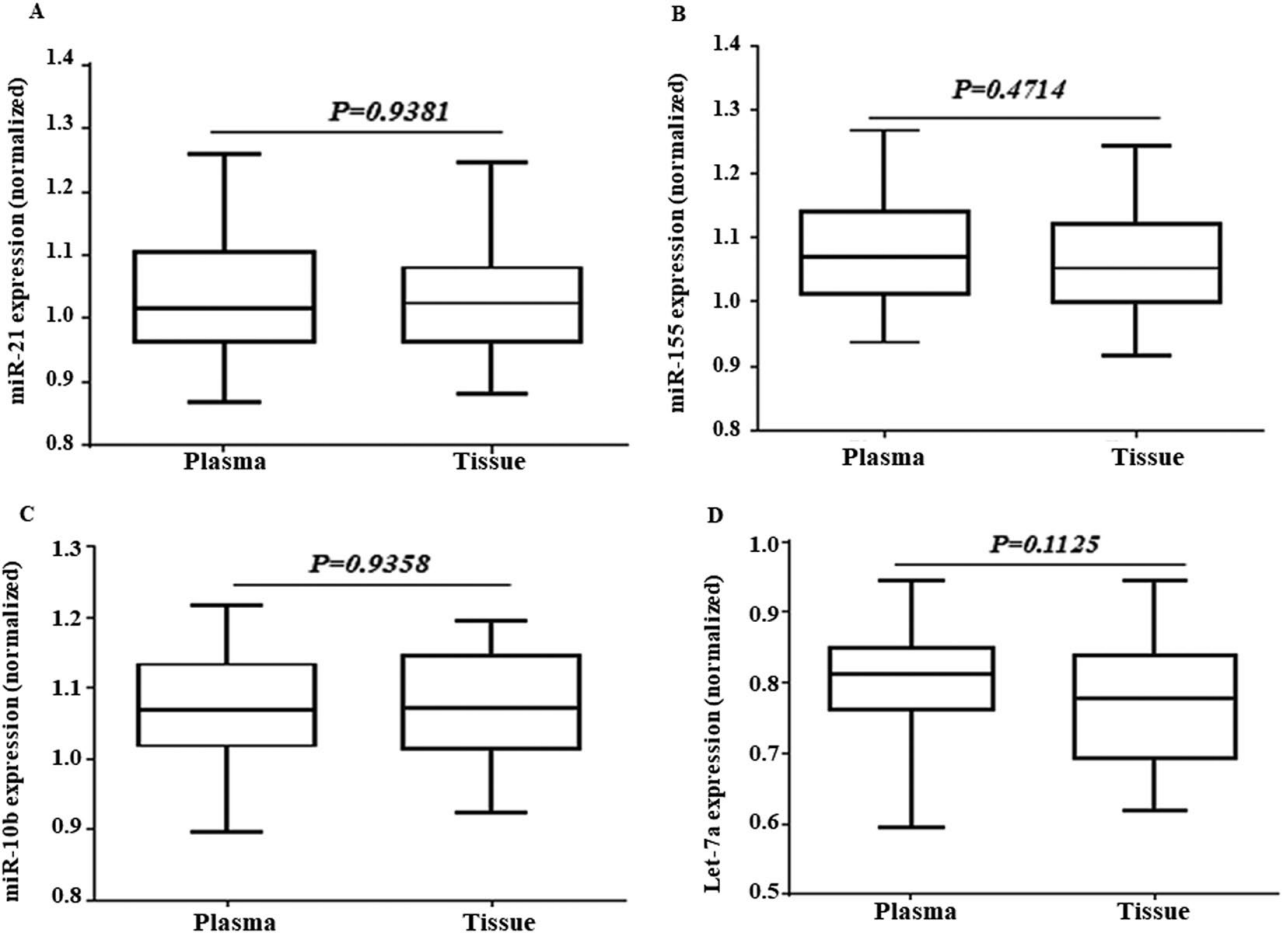
The differential expression of the selected miRs in both the tissue and plasma of the breast cancer patients. The relative expression level of the miR-21 (**A**), miR-155 (**B**), miR-10b (**C**), and Let-7a (**D**) were normalized using SNORD RNA as reference RNA. The line represents the median value.

Then, we have compromised the expression levels of the miR-21, miR-10b, miR-155, and Let-7a between the tissue and the plasma samples in 30 patients. The results showed a similar expression pattern between them and there were no significant changes (Fig. 2).

**Figure 3:**
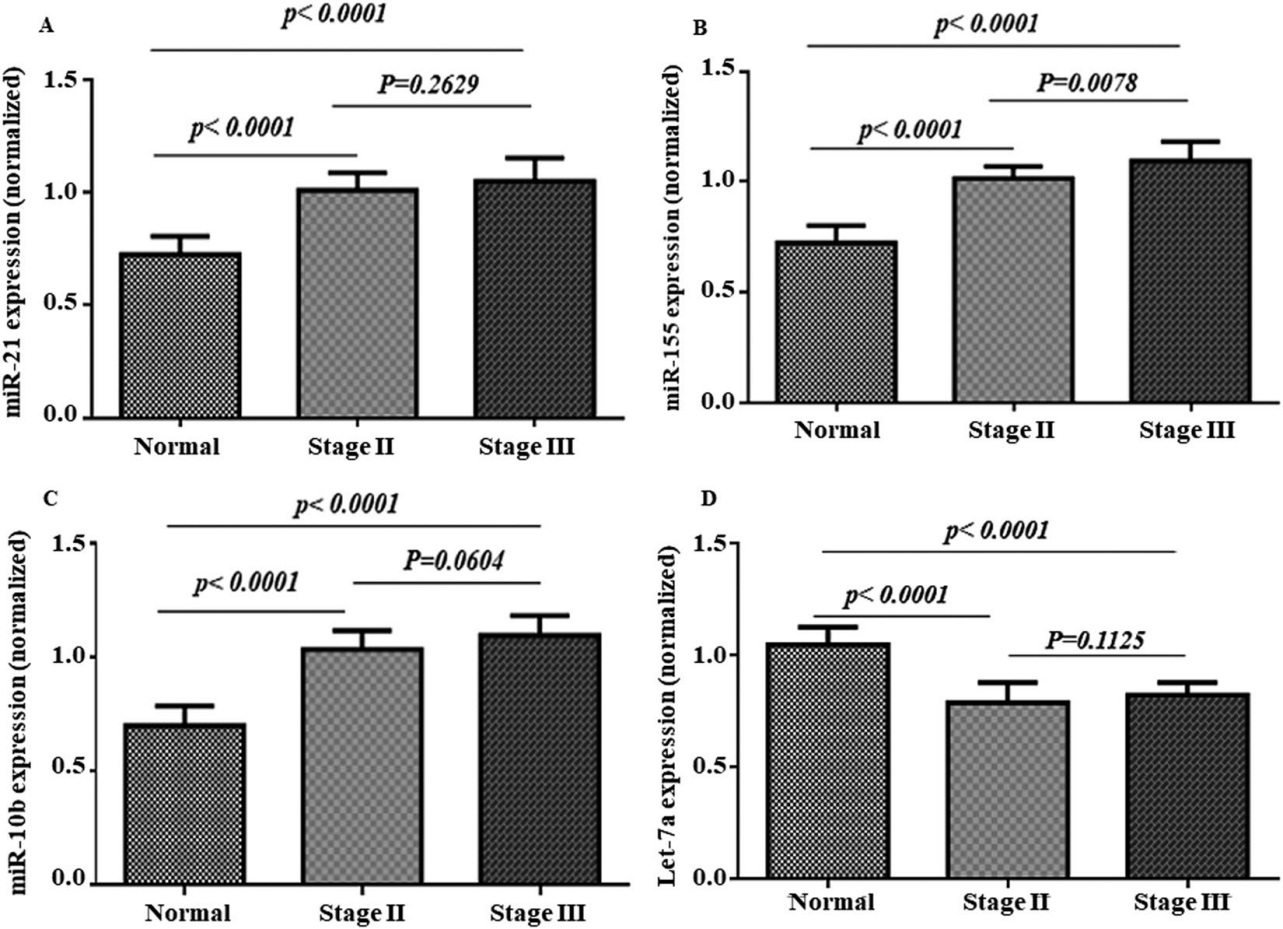
Comparison of stage II/III feature of the patients with the plasma relative expression of the selected miRs. The relative expression level of the miR-21 (**A**), miR-155 (**B**), miR-10b (**C**), and Let-7a (**D**) were normalized using SNORD RNA as reference RNA.

Moreover, we have stratified the patients to examine the associations between the plasma levels of the designated miRs and the stage II/III of the disease based on the TNM staging. Of the 30 cases of the breast cancer, the miR-21, miR-10b, miR-155, and Let-7a did show a significant difference in the staging compromised to the control (Fig. 3). Likewise, the expression of the miR-155 in the plasma showed a significant difference between stage II and III (p = 0.0078) (Fig. 3).

Furthermore, we have also compromised the expression levels of the miR-21, miR-10b, miR-155 and Let-7a between the tissue and plasma samples of the patients with the other clinical parameters including the nodal (Table 2) and menopause status (Table 3). The results showed a similar expression pattern between the nodal status (−)/(+) of miR-21, but there was a significant change in the nodal status of the plasma expression of miR-155 and miR-10b and the tissue expression of Let-7a (Table 2).

**Table 2:** The expression of the selected miRs and the nodal status (−)/(+) of the disease between the tissue and plasma samples.

Samples	Plasma		P-value	Tissue		P-value
miRs	Nodal status (−)	Nodal status(+)		Nodal status (−)	Nodal status (+)	
miR-21	1.0 ± 0.010	1.05 ± 0.02	0.1481	1.03 ± 0.03	1.03 ± 0.02	0.9148
miR-155	1.01 ± 0.01	1.091 ± 0.02	*0.0091	1.1 ± 0.03	1.07 ± 0.01	0.3637
miR-10b	1.02 ± 0.02	1.1 ± 0.02	*0.0218	1.07 ± 0.02	1.07 ± 0.01	0.9512
Let7a	0.8 ± 0.02	0.9 ± 0.01	0.8519	0.8 ± 0.03	0.7 ± 0.02	*0.0139

**Table 3:** Comparison of the menopause status of the breast cancer patients with the plasma relative expression of the selected miRs.

Samples	Plasma		P-value	Tissue		P-value
miRs	Pre	Post		Pre	Post	
miR-21	1.04 ± 0.02	1.03 ± 0.02	0.7684	1.04 ± 0.02	1.03 ± 0.03	0.7516
miR-155	1.06 ± 0.02	1.06 ± 0.02	0.8804	1.06 ± 0.02	1.1 ± 0.02	0.2644
miR-10b	1.1 ± 0.03	1.06 ± 0.02	0.5969	1.06 ± 0.02	1.08 ± 0.02	0.3689
Let7a	0.9 ± 0.01	0.8 ± 0.1	*0.0215	0.8 ± 0.1	0.8 ± 0.02	0.3233

Besides, our data showed a similar expression pattern between the menopause status of the miR-21, miR-10b, and miR-155 in both the plasma and tissue samples; nevertheless, there was a significant change in the menopause status of the Let-7a plasma expression (Table 3).

### Monitoring of the miR-21, miR-10b, miR-155, and Let-7a during the course of the treatment

**Figure 4:**
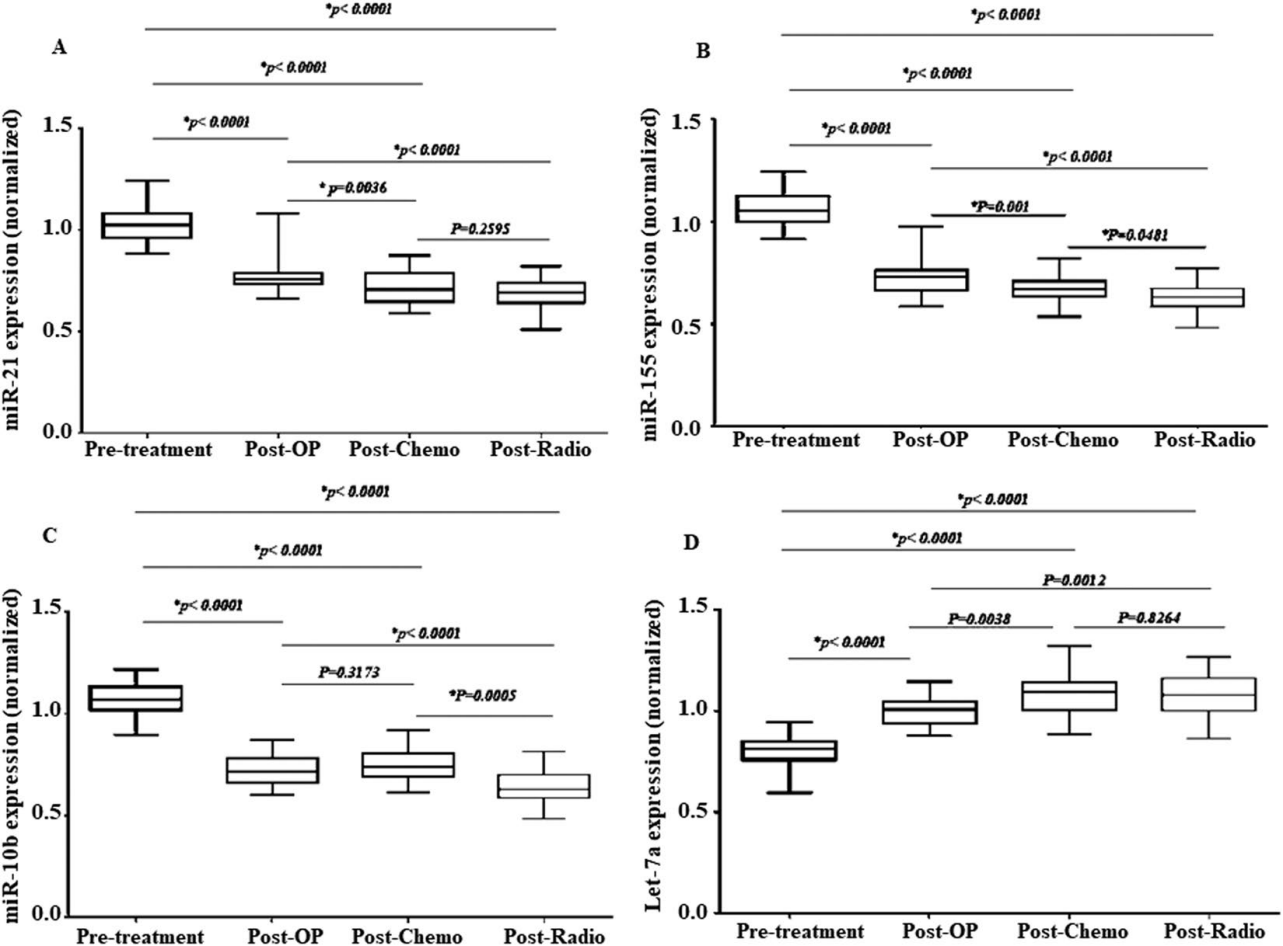
The expression of the selected miRs in the breast cancer patients before and after the treatment. The relative expression level of the miR-21 (**A**), miR-155 (**B**), miR-10b (**C**), and Let-7a (**D**) were normalized using SNORD RNA as reference RNA. The line represents the median value. Post-OP: Post-Operation, Post-Chemo: Post-chemotherapy, Post-Radio: Post-Radiotherapy.

Thirty patients with the non-metastatic breast cancer were monitored for changes in the plasma level of the miR-21, miR-10b, miR-155 and Let-7a before and after the surgery, chemotherapy, and radiotherapy. Then, we have compromised the expression levels of the miRs before and after the treatment. The miR-21 plasma level was significantly down-expressed after the operation (0.78 ± 0.09, p < 0.0001), chemotherapy (0.71 ± 0.08, p < 0.0001) and radiotherapy (0.70 ± 0.07, p < 0.0001) than the pre-treatment (1.03 ± 0.09) (Fig. 4A). There was a significant difference in its plasma level after the chemotherapy and radiotherapy than the post-operation (1.03 ± 0.09), but there was no significant difference between the post-chemotherapy and post-radiotherapy (0.02347) (Fig. 4A).

Moreover, the miR-155 plasma level was significantly down-regulated after the operation (0.72 ± 0.07, p < 0.0001), chemotherapy (0.66 ± 0.07, p < 0.0001) and radiotherapy (0.62 ± 0.06, p < 0.0001) than the pre-treatment (1.08 ± 0.08) (Fig. 4B). Interestingly, there was a significant difference in its plasma level after the operation, chemotherapy, and radiotherapy than each other (Fig. 4B).

Furthermore, the miR-10b plasma level was significantly decreased after the operation (0.72 ± 0.07, p < 0.0001), chemotherapy (0.75 ± 0.08, p < 0.0001) and radiotherapy (0.64 ± 0.07, p < 0.0001) than the pre-treatment (1.07 ± 0.09) (Fig. 4C). Additionally, there was no significant difference in its plasma level after the operation than the post-chemotherapy (p = 0.317), but there was a significant difference in its plasma level after the radiotherapy than the post-operation (0.72 ± 0.07, p < 0.0001) and post-chemotherapy (0.75 ± 0.08, p < 0.0001) (Fig. 4C).

The Let-7a plasma level was significantly up-expressed after the operation (1.0 ± 0.07, p < 0.0001), chemotherapy (1.08 ± 0.11, p < 0.0001) and radiotherapy (1.1 ± 0.10, p < 0.0001) than the pre-treatment (0.80 ± 0.07) (Fig. 4D). Nonetheless, there was no significant difference in its plasma level after the surgery, chemotherapy, and radiotherapy than each other (Fig. 4D).

**Figure 5:**
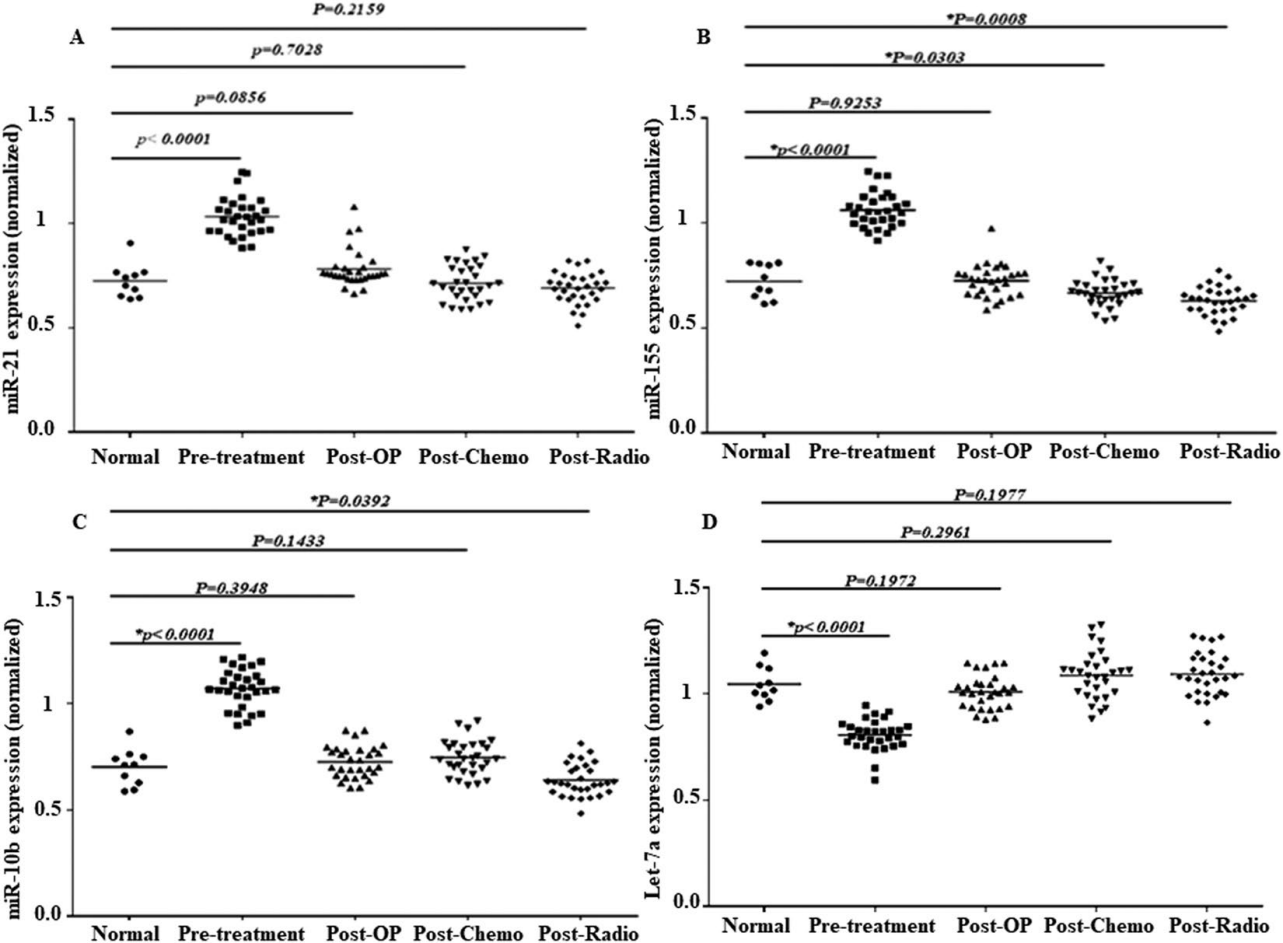
The expression of the selected miRs in the patients before and after the treatment compromised to the control. The relative expression of the miR-21 (**A**), miR-155 (**B**), miR-10b (**C**), and Let-7a (**D**) were normalized using SNORD RNA as reference RNA. The line represents the median value. Post-OP: Post-Operation, Post-Chemo: Post-chemotherapy, Post-Radio: Post-Radiotherapy.

Finally, we have compromised the plasma levels of the miRs between the control subjects and the course of treatments individually (Fig. 5). The miR-155 plasma level underwent more decrease post-chemotherapy (p = 0.03) and post-radiotherapy (p = 0.0008) than the control subjects. In addition, the miR-10b plasma level was done a more decrease post-radiotherapy (p = 0.0392) than the control subjects.

## Discussion

The aim of the present study is to evaluate the plasma levels of the miR-21, miR-10b, miR-155 and Let-7a in the non-metastatic BC patients following the common treatments such as the surgery, chemotherapy, and radiotherapy using RT qPCR. Our results showed that the expression levels of the oncomiRs such as the miR-21, miR-10b, and miR-155 were significantly increased, while the expression level of the tumor suppressor such as the Let-7a was significantly decreased in the plasma of the patients. Remarkably, using the common treatments has reversed these effects. In this context, many of the studies have been conducted on the expression of the miRs in the tumorigenesis processes^17^. The first report of the miRs related to cancer was shown in the patients with B cell chronic lymphocytic leukemia^18^. Blenkiron *et al*.^10^ have also identified 133 miRs that displayed the abnormal expression levels in the breast tumor tissues compromised to the normal breast tissues. Similar to our results, they showed a difference of 29 miRs which have a key role in breast cancer development^10^. Furthermore, we have compromised the expression levels of the miR-21, miR-10b, miR-155, and Let-7a between the tissue and the plasma samples of the patients. The results showed a similar expression pattern between them and there were no significant changes. This finding was not supported by the obtained results by Matamala *et al*.^9^. Nevertheless, Svoronos *et al*.^13^ have also shown that a high correlation between the miRNA expression level that was found between the breast tumor tissues and the serum level. Here, the miR-21, miR-106a, and miR-155 were significantly over-expressed in the tumor specimens compromised with the control, whereas miR-126, miR-199a, and miR-335 were significantly under-expressed. Furthermore, the relative expression of the miR-21, miR-126, miR-155, miR-199a, and miR-335 was closely associated with the clinicopathologic features of the breast cancer such as the histological tumor grades and expression of sex hormone receptor^13,19^.

We have also analyzed the results from the selected miRs expression in the plasma to evaluate whether there was a correlation between the expression level of the miRs and the various clinic-pathologic features or not. For example, the expression of the miR-21 and miR-155 is related to the expression of the estrogen receptor (ER) and progesterone receptor (PR)^20^. Approximately 70% of breast tumors overexpress ER^20^. The ER up-regulation during the early stages of tumorigenesis has been identified as an important factor in stimulating the mammary cell proliferation which can lead to tumor development^21^. Similarly, Al-Khanbashi *et al*.^22^ observed the expression patterns of the miRs associated with Her2/neu/ER/PR in the breast tumors^22^. In this context, two other independent studies have also shown an increase in the expression of the miR-21 in the breast cancer patients as compromised to the healthy ones^23^. Furthermore, the plasma levels of the miR-10b and miR-155 can also be detected between the breast cancer patients and the healthy ones. Besides, the serum level of the miR-155 in PR+ tumors has shown a significant difference in compromised to PR- cases^20^. Wang *et al*. (2015) showed that the expression of the miR-21 and miR-155 was associated with the clinical pathological features of the breast tumors such as the histological grades and the sex hormone receptor^24^. The molecular subtypes of the ER are characterized by different responses to the therapy, differential course, and prognosis^20^. The variances between the ER+ and ER− breast tumor not only relate to their morphology but also are mostly due to the alteration in their transcriptional reactions. Moreover, the expression of the oncomiRs can be due to the transcriptional exacerbation of their genes due to the availability of the transcription factors, hyper-methylation or placement in the intra-region or between the genes. MiRs are heavily linked to cancer and can play a role through the effects of the key points in cell cycle regulation, genome integrity, and response to stress, apoptosis, and metastasis. In this respect, the difference between the expressions of the miRs may be due to differences in the sample sources, different analytical methods, or different platform in the studies. Therefore, it seems that an increasing or decreasing expression of miRs can be related to various reasons.

Moreover, we have stratified the patients to examine the associations between the plasma levels of the designated miRs and the stage II/III of the disease based on the TNM staging. Of the 30 cases of the breast cancer, the miR-21, miR-10b, miR-155, and Let-7a did show a significant difference in the staging compromised to the control. Besides, the expression of the miR-155 in the plasma showed a significant difference between stage II and III. Wang *et al*. (2015) have shown that the miR-21, miR-106a, and miR-155 were significantly up-regulated in higher malignancy grades compromised to the control breast tissues^13^. The relative expression of the miR-21 was not altered in the benign tumors but increased 2 fold in the grade II tumors and 4.5 fold in the grade III tumors. MiR-155 expression was not altered in the benign tumors but was increased 2 fold and 5 fold in the grade II and III, respectively. In contrast, the miR-21, miR-126, miR-155, miR-199a, and miR-335 were highly correlated to ER or PR in both the grades. There was a greater difference in expression levels of samples with negative hormone receptor expression (P = 0.05), which was a predictive factor for prognosis of patients with breast cancer. The findings suggest that the miRs can be used to identify the different nature of breast tissues, and deregulation of the selected miRs may affect critical molecular events involved in tumor progression. Thus, the measurements of miRs as the biochemical markers can help to diagnose the different stages of cancer prior to clinical investigations on the samples.

Additionally, the present study investigates the changes in the expression level of the miRs after common treatments including operation, chemotherapy, and radiotherapy. Our results showed that the miR-21 plasma level was significantly down-expressed after the operation, chemotherapy, and radiotherapy than the pre-treatment. Moreover, there was a significant difference in its plasma level after chemotherapy and radiotherapy than post-operation. These results are consistent with Chang *et al*.^25^, Badr *et al*.^26^, and Farsinejad *et al*.^27^. They found that patients with better miR-21 expression after treatment have better clinical outcomes that can increase survival^25–27^. Studies have also shown that miR-21 can reduce the expression of the tumor suppressor proteins and increase the expression of the oncogene proteins^28^. Likewise, the increase in the expression of the miR-21 in the breast tumor tissues has been shown to be directly related to the incidence of the disease, tumor size, and staging. The miR-21 can be an effect on tumor cells and may be of an anticoagulant and anti-apoptosis effect, and disrupts the pathway of apoptosis which is in the interest of cancer cell survival^29^. Therefore, it seems that miR-21 can be a good alternative to monitoring cancer or metastasis.

Moreover, the present study showed that the miR-155 plasma level was significantly down-regulated after the operation, chemotherapy, and radiotherapy than the pre-treatment. Interestingly, there was a significant difference in its plasma level after the operation, chemotherapy, and radiotherapy than each other. Similar to our results, Sochor *et al*.^30^ showed that after surgery, the expression of the miR-155 was significantly decreased compromised to after chemotherapy. Their results have indicated a correlation between the removal of the breast tumors during the treatment and the miR-155 serum level^30^. Nevertheless, Sun *et al*.^31^ have shown a short-term increase in the expression of the miR-155 after surgery, probably due to the removal of the tumor and the miR-155 release to the blood, although the decrease of the mir-155 expression after chemotherapy was similar to our results^31^.

Additionally, the miR-10b plasma level was significantly decreased after the operation, chemotherapy, and radiotherapy than the pre-treatment. There was no significant difference in its plasma level post-operation than post-chemotherapy, but there was a significant difference in its level of post-radiotherapy than post-operation and post-chemotherapy. Ma *et al*.^32^ showed that miR-10b level in the non-metastatic breast tumors was declined in the metastatic patients^32^. Similar to our results, Gee *et al*.^33^ showed that the decreased levels of the miR-10b expression can occur in the early stages of the breast tumors^33^. Moreover, our results showed a significant difference in the expression level of mir-10b after the operation, chemotherapy, and radiotherapy which is contrary to the results of Iorio and Croce *et al*.^34^. In addition, a meta-analysis by Huang *et al*.^35^ showed that the expression of the miR-10b is different in the tumor stages. These differences may be related to the cell type, the tumor stage or the tissue source^35^. Thus, the miR-10b can be a prognosis for the early detection or a therapeutic goal for metastasis treatment.

In addition, our results showed that the Let-7a plasma level was significantly up-expressed after the operation, chemotherapy, and radiotherapy than the pre-treatment which is similar to Weidhaas *et al*.^36^. Wang *et al*. (2013) have also shown that the high expression of the Let-7 can increase the sensitivity of the breast cancer cells to radiotherapy, which can help with treatment^37^. Several studies have shown the Let-7 variations in the sensitivity of the breast cancer cells during radiotherapy^37^. Surprisingly, in the present study after radiotherapy, the Let-7a level was increased and reached the level of the healthy ones.

## Conclusion

In summary, the expression of the miR-21 and miR-155 was decreased, and the expression of Let-7a was increased at the end of the present study and reached the expression level of healthy individuals. The chemotherapy and radiotherapy were led to a more reduction in the oncomiRs and an increase of the tumor suppressor. Generally, in the absence of a recognizable tumor, an increase in the oncomiRs level or a decrease in the tumor suppressors may indicate a failure in treatment. As a result, measuring the expression of the miRs after the operation, chemotherapy, and radiotherapy can be considered as a recognizable marker for proper response of the patients to the treatment. It is worth noting that reducing oncomiRs and increasing tumor suppressors during treatment can be considered as a good diagnostic tool for the process of improvement and proper response to the standard treatments. Moreover, it should be noted that we will follow the patients annually for the first 5 years to relapse the disease or metastasis using the liquid biopsy and measure the candidate miRs.

## Materials and Methods

### Ethics statement

This study was approved by the Medical Ethics Committee of the Tehran University of Medical Sciences (NO: 23797) and Iranian Randomized Control Trial (IRCT) ethical board. In addition, the written informed consent was obtained from each participant prior to the sample collection.

### Study design and sample collection

In the present study, the primary breast tumor samples were obtained from the 30 patients (Luminal A; ER^+^, PR^+^, Her2-) with informed consent approved, and the level of the plasma miR-155, miR-21, miR-10b, and Let-7a in the cases and 10 control plasma samples were screened. Then, we evaluated the changes in the levels of the miRs after the operation, chemotherapy, and radiotherapy. Pre-operative plasma from the patients with the histologically diagnosed breast cancer (n = 30) was drawn at Imam Khomeini Hospital, Cancer Institute of Iran from Oct 2016 to Sep 2017. The characteristic of the patients including age, T classification, nodal status, hormone-receptor positive, HER2 overexpression, and tumor subtype were retrospectively collected (Table 4). The patients with the severe infection, active clinical comorbidities, or a history of any other malignancy were excluded. For 30 patients who underwent treatment, the second sample was obtained one week before the chemotherapy, the third sample was collected at the periodical evaluation one week before the commencement of radiotherapy, and the fourth sample was collected at the periodical evaluation one month after radiotherapy. The applied adjuvant chemotherapies were epirubicin/cyclophosphamide, epirubicin/taxane, cyclophosphamide/pirarubicin or fluorouracil epirubicin/cyclophosphamide with and without taxane. In these patients, the response to therapy was assessed by the specialist doctors according to the World Organization (WHO) guidelines^38^. Additionally, the plasmas from a set of the 10 healthy females were collected from outpatients at Imam Khomeini Hospital. All participants were of Iranian ethnic. None of the healthy controls had previously diagnosed with any malignancies. The median age of these healthy cases was 45 (range from 26 to 70). There is no significant difference in age between the breast cancer patients and the controls (p = 0.6999, Mann-Whitney t-test). The blood sample from each participant was collected in the tube with EDTA (BD vacutainer SSTTM Tubes, Reference No. 367985). After exposure to the room temperature for 30 min to 2 hours, the specimens were centrifuged at 1,500 g for 20 min at 4 °C. The plasmas were aliquoted into microcentrifuge tubes and stored at −80 °C before use.

**Table 4:** The information of the patients.

Characteristic	Patients (%)
Total	N = 30
**Age**	
Mean	45.52 years
Median (range)	45.5 (range 26–70 years)
**TNM stage**	
II	13
III	17
**T classification**	
T1	7
T2	11
T3	12
**Nodal status**	
Negative	11
Positive	19
**ER**^**+**^, **PR**^**+**^, **Her2**^**−**^	
Negative	0
Positive	30
**Menopause**	
premenopausal	14
postmenopausal	16
**Subtype**	
Luminal A	30

### Identification of the breast cancer-related miRs

The Gene Expression Omnibus database (GEO, http://www.ncbi.nlm.nih.gov/geo/) in the National Center for Biotechnology Information (NCBI) is the largest fully public gene expression resource and includes 214,268 samples and 4,500 platforms^39^. The selected miRs were mapped into the human miR disease database (HMDD; http://cmbi.bjmu.edu.cn/hmdd and http://202.38.126.151/hmdd/tools/hmdd2.html) to further select the differentially expressed miRs related to BC. As a database for experimentally supported human miRs and disease associations, HMDD serves as a valuable resource for studying the roles of miRs in human disease^40^. Furthermore, the target genes of the differentially expressed miRs of breast cancer-related tissues were predicted by five miR databases, named miRanda (http://microrna.sanger.ac.uk)^41^, MirTarget2 (http://nar.oxfordjournals.org/cgi/content/abstract/34/5/1646)^42^, PicTar (http://pictar.bio.nyu.edu)^43^, PITA (http://genie.weizmann.ac.il/pubs/mir07)^44^ and TargetScan (http://targetscan.org)^45^. Additionally, the published oncogenes and suppressors of breast cancer were selected from TSGene (http://bioinfo.mc.vanderbilt.edu/TSGene/)^45^ and Tumor-Associated Gene (TAG; http://www.binfo.ncku.edu.tw/TAG/) databases^46^. In the present study, DAVID has applied to conduct Kyoto encyclopedia of genes and genomes (KEGG) pathway and gene ontology (GO) enrichment analyses for the identified target genes. KEGG is a knowledge base for systematic analysis of gene functions^47^.

### Quantitative Real-Time PCR Analysis

Total RNA was extracted from 100 µl plasma samples and 50 gr tumor samples using 1 ml trizol reagent according to the manufacturer’s instructions (Sinagene, Tehran, Iran). The trizol reagent is used for isolating both enriched miRNAs and larger RNA species. Qualitative and quantitative assessment of the isolated RNA was carried out by the electrophoresis and spectrometric methods^48^. The RNA was stored at −80 °C for later analysis. For miRs quantification by Real-Time PCR in all samples, 10 µl of the total RNA were reverse-transcribed in a 20 µl reaction mix using the BONmiR 1st-strand cDNA synthesis kit (stem cell technology research center, Tehran, Iran) following the manufacturer’s recommendations. Then, cDNA was used in each of the real-time PCR assays with the BONmiR qPCR Kit (stem cell technology research center, Tehran, Iran) based on the manufacturer’s instructions. Real-Time PCR analyses of the miRs were carried out in triplicate. The levels of miRs were normalized using SNORD RNA as reference RNA. MicroRNA gene expression was analyzed by means of the Step-One system (ABI, Massachusetts, USA) and the expression levels were evaluated using 2^(−∆∆ct)^.

Relative expression of the miRNA was normalized to SNORD and was calculated using the 2^(−∆∆ct)^ method. ΔCT was calculated by subtracting the CT values of SNORD from the CT values of the target miRs. ΔΔCT was then determined by subtracting average ΔCT of the control from the ΔCT of cases. The fold changes of candidate miRNA expression were calculated by the equation 2^(−∆∆ct)^^49,50^.

The expression levels were compromised with the healthy ones and expressed as fold change. Sequences of the used forward primers are as below:

MiR-21 Forward primer: ACGTGTTAGCTTATCAGACTG

MiR-155 Forward primer: CCGTTAATGCTAATCGTG

MiR-10b Forward primer: TAAGCACGAGACTTACGGAGGA

Let-7a Forward primer: GGCTGAGGTAGTAGGTTGTATAG

Snord Forward primer: ATCACTGTAAAACCGTTCCA

Universal Reverse Primers were obtained from Bonyakhteh Company (Bonyakhteh, Tehran, Iran)

### Data analysis

All data presented as mean ± SD. The statistical analyses were performed using the SPSS 16.0 software (SPSS) and the GraphPad Prism 5.0, GraphPad. Kolmogorov-Smirnov was used to evaluate the natural distribution of data. For inferential analysis of data in two groups, parametric data from the t-test and non-parametric data from the Mann-Whitney method were used. The repaid measured ANOVA test was used to evaluate the intra-group treatment trend. P values less than 0.05 was considered to be statistically significant.

### Ethical approval

All procedures performed in studies involving human participants were in accordance with the ethical standards of the institutional and/or national research committee and with the 1964 Helsinki declaration and its later amendments or comparable ethical standards.

### Informed consent

Informed consent was obtained from all individual participants included in the study.
